# Alginate-Based Biomaterials for Regenerative Medicine Applications

**DOI:** 10.3390/ma6041285

**Published:** 2013-03-26

**Authors:** Jinchen Sun, Huaping Tan

**Affiliations:** School of Materials Science and Engineering, Nanjing University of Science and Technology, Nanjing 210094, China; E-Mail: achen11@sina.cn

**Keywords:** biomaterials, alginate, regenerative medicine, tissue engineering, drug delivery

## Abstract

Alginate is a natural polysaccharide exhibiting excellent biocompatibility and biodegradability, having many different applications in the field of biomedicine. Alginate is readily processable for applicable three-dimensional scaffolding materials such as hydrogels, microspheres, microcapsules, sponges, foams and fibers. Alginate-based biomaterials can be utilized as drug delivery systems and cell carriers for tissue engineering. Alginate can be easily modified via chemical and physical reactions to obtain derivatives having various structures, properties, functions and applications. Tuning the structure and properties such as biodegradability, mechanical strength, gelation property and cell affinity can be achieved through combination with other biomaterials, immobilization of specific ligands such as peptide and sugar molecules, and physical or chemical crosslinking. This review focuses on recent advances in the use of alginate and its derivatives in the field of biomedical applications, including wound healing, cartilage repair, bone regeneration and drug delivery, which have potential in tissue regeneration applications.

## 1. Introduction

Regenerative medicine, which combines tissue engineering and drug delivery, utilizes the multidisciplinary principles of materials science, medicine, and life science to generate tissues and organs of better biological structures and functions. Regenerative medicine is to implant scaffolding materials for regenerating tissue based on the recruitment of native cells into the scaffold, and subsequent deposition of extracellular matrix (ECM). Cell scaffolds play a crucial role because they act as an artificial ECM to provide a temporary environment to support the cell to infiltrate, adhere, proliferate and differentiate [1,2,3]. Cell scaffolds provide the initial structural support and retain cells in the defective area for cell growth, metabolism and matrix production, thus playing an important role during the development of engineered tissues [4].

For an ideal scaffolding material, properties are required that include biocompatibility, suitable microstructure, desired mechanical strength and degradation rate as well as most importantly the ability to support cell residence and allow retention of metabolic functions [5,6]. Various natural and synthetic biomaterials have been considered as cell supporting matrices. Polymers of natural origin are attractive options, mainly due to their similarities with ECM as well as their chemical versatility and biological performance.

**Figure 1:**
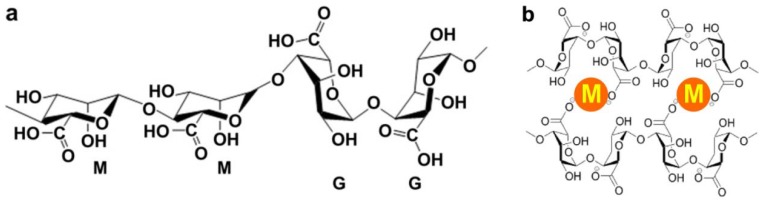
(**a**) Chemical structure of alginate; (**b**) Mechanism of ionic interaction between alginate and divalent cations.

Alginate is a naturally occurring anionic and hydrophilic polysaccharide. It is one of the most abundant biosynthesized materials [7,8], and is derived primarily from brown seaweed and bacteria. Alginate contains blocks of (1–4)-linked *β*-D-mannuronic acid (M) and *α*-L-guluronic acid (G) monomers (Figure 1a). Typically, the blocks are composed of three different forms of polymer segments: consecutive G residues, consecutive M residues and alternating MG residues.

Alginate is of particular interest for a broad range of applications as a biomaterial and especially as the supporting matrix or delivery system for tissue repair and regeneration. Due to its outstanding properties in terms of biocompatibility, biodegradability, non-antigenicity and chelating ability, alginate has been widely used in a variety of biomedical applications including tissue engineering, drug delivery and in some formulations preventing gastric reflux [9,10]. To chelate with divalent cations is the easiest way to prepare alginate hydrogels from an aqueous solution under gentle conditions (Figure 1b). As a result of the naturally occurring polysaccharide, alginate exhibits a pH-dependent anionic nature and has the ability to interact with cationic polyelectrolytes and proteoglycans. Therefore, delivery systems for cationic drugs and molecules can be obtained through simple electrostatic interactions.

Scaffolds are often used for the delivery of drugs, growth factors and therapeutically useful cells. As such, scaffolding materials allow protection of biologically active substances or cells from the biological environment. Depending on the site of implantation, the biomaterials are subjected to different pH environments, which affect the degradation properties, mechanical properties and swelling behaviour of the biomaterials. As such, alginate plays an important role in the long term stability and performance of alginate-based biomaterials *in vitro*. The molecular weight (MW) of alginate influences the degradation rate and mechanical properties of alginate-based biomaterials. Basically, higher MW decreases the number of reactive positions available for hydrolysis degradation, which further facilitates a slower degradation rate. In addition, degradation also inherently influences the mechanical properties owing to structural changes both at molecular or macroscopic levels.

As a U.S. Food and Drug Administration (FDA)-approved polymer, alginate has become one of the most important biomaterials for diverse applications in regeneration medicine, nutrition supplements, semipermeable separation etc. [11,12,13,14,15]. This review focuses on the most important biomaterial forms, e.g., hydrogels, microspheres, porous scaffolds and fibers, fabricated from alginate and its derivatives. Particularly, the modification of the alginate molecule and the process method to obtain the desired properties and functions is introduced. The applications of alginate-based materials for repair and regeneration of various tissues and organs such as skin, cartilage and bone are summarized.

## 2. Major Systems

### 2.1. Hydrogels 

Hydrogels are three-dimensionally cross-linked networks, which are composed of hydrophilic polymers with high water content [16,17,18,19,20]. When cells are incorporated into hydrogels, their highly swollen state facilitates transport of nutrients into and cellular waste out of the hydrogels [19,20,21,22,23,24,25]. Additionally, a general advantage of injectable hydrogels is the utilization of minimally invasive surgery as compared to open surgery [20,25,26,27,28]. Generally, alginate is hydrophilic and water-soluble, thickening in neutral conditions, which is of great importance for *in situ* hydrogel formation. Alginate hydrogels with potential applications in tissue engineering can be classified into physical and covalent gels, according to their gelation mechanisms. Many methods have been employed for preparation of alginate hydrogels, including ionic interaction, phase transition (thermal gelation), cell-crosslinking, free radical polymerization and “click” reaction [1,17]. Basically, alginate hydrogels are likely to show pH responsive properties due to the presence of carboxyl groups on the backbone. The pH responsive behavior is evident from higher swelling ratios at increasing pH values due to chain expansion from the presence of ionic carboxylate groups on the backbone. Since alginate lacks informational structure for positive cell biological response, modification of synthetically derived alginate hydrogels is usually required.

#### 2.1.1. Ionic-Crosslinking

The most common method to prepare alginate hydrogels from an aqueous solution is to combine the alginate with divalent cations, ionic crosslinking agents [29,30]. In the presence of divalent cations, simple gelation can occur when divalent cations cooperatively interact with blocks of G monomers to form ionic bridges (Figure 1b). In a solution of alginate, blocks of M monomers form weak junctions with divalent cations. However, the interactions between blocks of G monomers and divalent cations form tightly held junctions.

Over the past decade, ionic cross-linked alginate hydrogels have been developed and employed in a variety of settings, such as with Ca^2+^, Mg^2+^, Fe^2+^, Ba^2+^, or Sr^2+^. Usually, Ca^2+^ is one of the most commonly used divalent cations used to ionically cross-link alginate and calcium chloride (CaCl_2_) is one of the best choices [10,31]. Ionically crosslinked alginate hydrogel disperses via an ion exchange process involving loss of divalent ions into the surrounding medium. However, the speed of gelation is too fast to be controlled due to the high solubility of calcium chloride in aqueous solution, which limits the application on injectable scaffolds. Also, the gelation speed affects gel uniformity and strength directly. In order to slow and control the gelation, CaCl_2_ can be replaced by calcium sulfate (CaSO_4_) or calcium carbonate (CaCO_3_) which have lower solubilities. Furthermore, ionically crosslinked alginate hydrogel has limited drug loading efficiency, strength and toughness, which limits its application in regenerative medicine [30,31]. Therefore, alginate has to be modified to improve its properties by other physical or chemical cross-linking methods.

#### 2.1.2. Phase Transition

**Figure 2:**
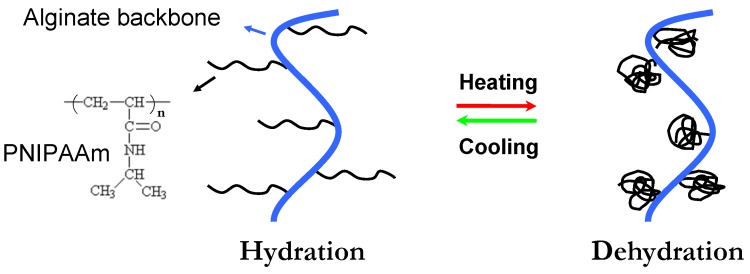
Schematic showing the temperature dependent behavior of PNIPAAm-g-alginate hydrogels. PNIPAAm = Poly(*N*-isopropylacrylamide)

Thermoresponsive phase transition has been utilized for hydrogel formation because gelation can be realized simply as the temperature increase above the lower critical solution temperature (LCST) [17]. Alginate hydrogels, capable of phase transition in response to external temperature, represent another way of preparing injectable scaffolds. Poly(*N*-isopropylacrylamide) (PNIPAAm) is well known for its ability to show LCST behavior in aqueous solutions at 32 °C [32,33,34,35]. The main mechanism of phase separation of PNIPAAm is thermally induced release of water molecules bound to the isopropyl side groups above its LCST, which results in increasing inter- and intra-molecular hydrophobic interactions between isopropyl groups [36,37,38,39,40,41]. The thermosensitivity of an alginate hydrogel can be achieved by incorporating PNIPAAm into its backbone. Figure 2 shows a schematic representing the temperature dependent behavior of PNIPAAm grafted alginate (PNIPAAm-g-Alginate) hydrogels. The procedure involves the synthesis of an amino-terminated NIPAAm copolymer (PNIPAAm-NH_2_), which is then covalently coupled with carboxyl groups (-COOH) of alginate involving water-soluble carbodiimide chemistry [33]. Temperature dependent behavior of PNIPAAm-g-Alginate hydrogels was evident from a noticeable decrease in the swelling ratio above 32 °C.

The other effective method to synthesize thermosensitive alginate hydrogel is combination with Pluronic F127. Pluronic F127 belongs to a class of block copolymers that consist of polyoxyethylene and polyoxypropylene, which also exhibit a thermoreversible gelation response. Pluronic F127 is one of the very few synthetic polymeric materials approved by the FDA for use in clinical applications. The potential drawbacks of Pluronic F127 are its weak mechanical strength and rapid erosion. In order to improve gelling properties, Pluronic F127 can be physically blended with alginate or chemically grafted onto alginate [42]. These modifications with alginate can improve the physical and mechanical properties of the thermo-reversible hydrogels.

Many reports have shown that thermoreversible alginate hydrogels that reversibly form a gel in response to the simultaneous variation of at least two physical parameters (e.g., pH, temperature, or ionic strength) can be blended to target their physical and mechanical properties [32,33]. The potential application of a thermo-responsive alginate hydrogel as a functional injectable cell scaffold in tissue engineering was studied by the encapsulation behavior of human stem cells, e.g., mesenchymal stem cells (MSCs) and adipose-derived stem cells (ASCs) [42].

#### 2.1.3. Cell-Crosslinking

**Figure 3:**
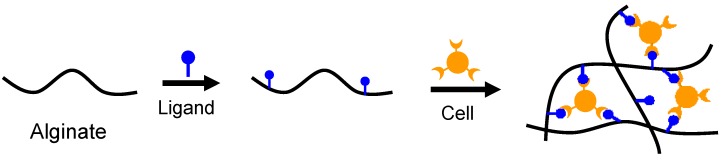
Schematic showing cell-crosslinked network formation of ligand modified alginate.

Specific receptor-ligand interactions have been employed to crosslink alginate hydrogels. Although it exhibits good biocompatibility, alginate is composed of inert monomers that inherently lack the bioactive ligands necessary for cell anchoring. The strategy of cell-crosslinking is to introduce ligands, e.g., arginine-glycine-aspartic acid (Arg-Gly-Asp, RGD) sequence onto alginate for cell adhesion by chemically coupling utilizing water-soluble carbodiimide chemistry [43,44,45]. Once mammalian cells have been added to this RGD-modified alginate to form a uniform dispersion within the solution, the receptors on the cell surface can bind to ligands of the modified alginate. The RGD-modified alginate solution has been subsequently cross-linked to form network structures via specific receptor-ligand interactions between cell surface and RGD sequences (Figure 3). Although the cell-crosslinked hydrogel shows excellent bioactivities, the network exhibits low strength and toughness, which may limit its practical applications.

#### 2.1.4. Free Radical Polymerization

Free radical polymerization means the process of transforming linear polymer into a three-dimensional polymer network, which can be carried out at physiological pH and temperature with the appropriate chemical initiators, even in direct contact with drugs and cells [46,47,48]. The mild gelation conditions allow cells to be encapsulated within radical polymerized hydrogels and remain viable. This can provide better temporal and spatial control over the gelation process. The unique advantage of chain polymerization is the ease with which a variety of chemistries can be incorporated into the hydrogel by simply mixing derivatized macromers of choice and subsequently copolymerizing [48,49,50,51,52,53].

**Figure 4:**
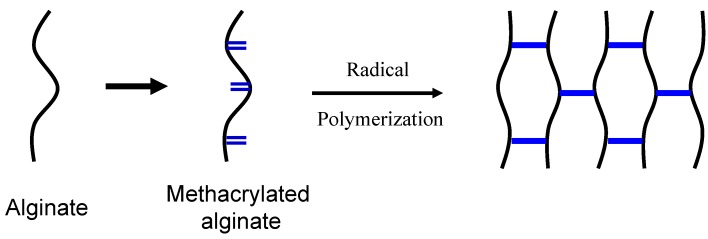
Schematic illustration of the preparation of methacrylated alginate and photocrosslinking of methacrylated alginate.

Many researchers have been interested in exploiting free radical polymerization of methacrylated alginate with unsaturated C=C double bond groups to create hydrogels as cell delivery vehicles for tissue regeneration (Figure 4). An extensively studied methacrylated alginate hydrogel is formed by employing ultraviolet (UV) irradiation to generate radicals from appropriate photoinitiators, which further react with the active end group on the methacrylated alginate to form covalent crosslinked bonds [54,55,56,57]. Since the photoinitiator could be harmful to the body in the process of photoinitiated polymerization, an appropriate photoinitiator should be selected to limit deleterious effects. The efficacy and biocompatibility of photopolymerization with 2-hydroxy-1-[4-(2-hydroxyethoxy) phenyl]-2-methyl-1-propanone (Irgacure 2959) as the initiator was demonstrated under irradiation with UV exposure [47]. The minimal cytotoxicity of Irgacure 2959 found over a broad range of mammalian cell types and species was indicated by previous researches [47,48,49,50,51].

In order to circumvent the injection problem in photopolymerization, methacrylated alginate can be covalently thermo-crosslinked to form a hydrogel at body temperature by initiation of a redox system, ammonium persulfate (APS) and *N,N,N’,N’*-tetramethylethylenediamine (TEMED). It was determined that the APS/TEMED initiation system is water-soluble and cytocompatible and thus can be used to initiate the polymerization of poly(propylene fumarate) (PPF) [56,57,58]. Previous studies have demonstrated that methacrylated alginate can be used to encapsulate chondrocytes and human ASCs with the APS/TEMED initiation system. Cell suspensions in methacrylated alginate solution can be injected into the body and polymerized at body temperature to form a crosslinked alginate gel that functions as a tissue scaffold [59]. Furthermore, copolymerization of methacrylated alginate with other synthetic macromers such as diacrylate and dimethacrylate enables additional control of functionality with properties that are especially important from a tissue engineering perspective. Hybrid artificial scaffolds that combine the physical characteristics of the alginate and bioactive features of other polymers can at the same time provide an ideal microenvironment for encapsulated cells.

#### 2.1.5. “Click” Reactions

Recent developments have utilized “click” reactions to prepare biodegradable hydrogels with specific association mechanisms, the most common example being 1,3-dipolar cycloadditions, the copper (I)-catalyzed reaction of azides with alkynes. While the versatility of metal-mediated “click” reactions has been broadly exploited, a major limitation is the intrinsic toxicity of transition metals and the inability to translate these approaches into regenerative medicine [28]. Since metal-free variants provide important alternatives, attempts have been devoted towards exploiting simple and highly efficient metal-free “click” conjugation.

**Figure 5:**
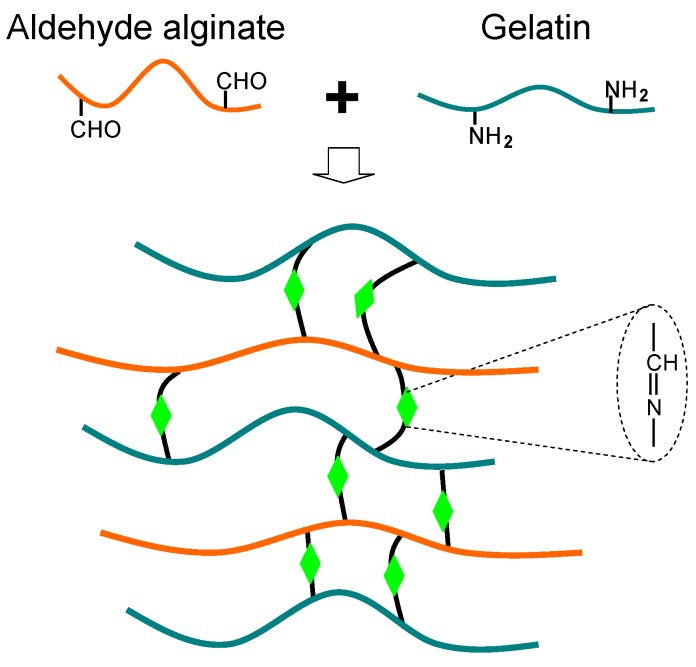
Scheme of alginate-gelatin composite hydrogel via the Schiff-base reaction.

A biocompatible and biodegradable alginate-gelatin composite hydrogel based on the biocompatible “click” reaction has been developed for tissue engineering applications [13,60]. The gelation is attributed to the Schiff-base reaction between aldehyde groups of oxidized alginate and amino groups of gelatin (Figure 5). The carbon-carbon bonds of the cis-diol groups in the molecular chain of the alginate can be cleaved to generate reactive aldehyde functions by periodate oxidation, which can develop chemical crosslinking with amino functions via Schiff-base linkage. In addition to gelatin, other biopolymers with amino groups such as chitosan and collagen can be employed for the Schiff-base linkage with oxidized alginate [61,62,63]. More recently, Krause *et al.* reported an aqueous metal-free “click” conjugation of a cyclic RGD-pentapeptide with alginate, creating a bioactive biomacromolecule [64]. These metal-free “click” conjugated alginates are applicable to a broad class of biodegradable scaffolds, without the need to employ any extraneous chemical crosslinking agents. They create a biomimetic microenvironment with improved biocompatibility and biodegradation for tissue regeneration.

A major issue is to design bioactive alginate-based hydrogels that would be readily injectable at or below room temperature, would form gels with relatively appropriate biodegradable properties under physiological conditions, and would support cell induction [65,66,67]. An ideal alginate hydrogel would potentially mimic many roles of ECM found in tissues, resulting in the coexistence of both physical and covalent gels. There is a continuing need to exploit novel crosslinking methods to enhance bioactive and mechanical properties of alginate hydrogels.

### 2.2. Microspheres 

Delivery systems based on microsphere technologies have been used to deliver cells, growth factors, proteins, genes and other drugs in tissue engineering [68,69,70,71]. Alginates can readily form gel- and solid-microspheres in the presence of suitable methods to be made as delivery systems. Basically, alginate gel-spheres are prepared under aqueous conditions via ionic crosslinking, and they are suitable for encapsulation of cells, growth factors and bioactive proteins [72,73,74,75,76,77,78]. Compared to the gel-spheres, alginate solid-spheres can be fabricated by emulsion solvent evaporation techniques, which are mainly to load drugs. Both alginate-based gel- and solid-microspheres show good biocompatibilities when they are used for regenerative medicine.

#### 2.2.1. Gel-Spheres

Although many synthetic microspheres have served as delivery systems, growth factors would be denatured and their bioactivities lost under the extreme preparation conditions when using organic solvents [72,73,74,75]. The organic solvent together with high shear stresses can induce denaturation and loss of biological activity of encapsulated growth factors and proteins. Generally, growth factors that are encapsulated in the aqueous and physiological environment can be more efficiently transported to a localized site and be released in a sustained-dosage form. The microencapsulation technique, an attractive approach to encapsulate and deliver cells or bioactive molecules, can provide a protective shell for live cells, cytokines, small proteins and other bioactive compounds [73,74,75,76,77]. As mentioned above, alginate solutions can quickly form hydrogels under mild conditions when exposed to divalent cations. Alginate gel-spheres, which are ionically crosslinked in the presence of Ca^2+^, have been used widely for the controlled delivery of cells and growth factors from aqueous fabrication conditions [76,77,78,79].

**Figure 6:**
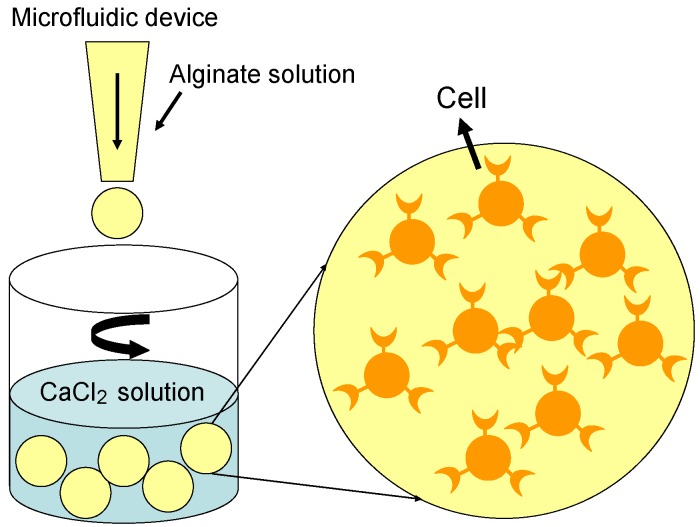
Illustration of the procedure for alginate gel-spheres in containing cells.

Cells or growth factors are carefully mixed evenly with the alginate solution, and the gel-microspheres are formed in an isotonic CaCl_2_ solution under constant stirring (Figure 6). The diameter of the alginate gel-microspheres lies between 200 µm and 500 µm, and the cells are distributed homogeneously inside the gel-microspheres [78,79,80,81,82]. For growth factor encapsulation, transforming growth factor-beta (TGF-*β*) is firstly combined with alginate solution to achieve a uniform solution, and then cross-linked with Ca^2+^ in CaCl_2_ solution to form gel-microspheres. Monodispersed alginate droplets can be generated to form uniform gel-spheres with consistent pore sizes by using a microfluidic device [80,81,82,83]. Besides, the uniform alginate gel-spheres can cumulate to highly organized 3D gel-sphere scaffolds with interconnecting porous structures [84]. The alginate gel-spheres are semi-permeable and have been shown to provide immune protection for many cell types and recipients, which allows cells to adhere, proliferate and differentiate. Furthermore, alginate gel-spheres enable high diffusion rates of macromolecules, which can be controlled to diffuse from the gel-microspheres at a high speed.

Simple alginate gel-spheres formed with divalent cations cannot sufficiently meet the needs of biological medicine due to limited encapsulation efficiencies. In a recent report, alginate was grafted with peptides containing a RGD sequence to promote cell adhesion [76]. The RGD-modified alginate gel-microspheres promote the ability of adhesion, proliferation, differentiation and enhance the mineralization potential of osteoprogenitor cells. More functionally, platelet-rich plasma (PRP) and ASCs were mixed and encapsulated together in alginate gel-microspheres [75]. The PRP-ASCs-laden alginate gel-microspheres were endued with osteogenic and angiogenic potential by combination of PRP and ASCs. The modified alginate can be utilized in the form of gel-spheres or colloidal particles to transport molecules through mucosa and epithelia because of their high affinity for the cell membranes.

#### 2.2.2. Solid-Spheres

Biodegradable microspheres and nanoparticles have been extensively used as drug carriers [85]. Biodegradable polymers such as poly(lactic acid) (PLA), poly(lactide-co-glycolide) (PLGA), chitosan, gelatin and alginate are now largely used to prepare microspheres and nanoparticles [2,69,70,71]. Generally, following intravenous injection, nanoparticles can be rapidly cleared from the blood by the mononuclear phagocyte system (MPS). Moreover, it is well known that the cells predominantly involved in this uptake are the macrophages of the liver, the spleen and circulating monocytes. The more hydrophobic the nanoparticle surface is, the more rapid is their uptake from circulation. This can be modulated by the particle size and surface properties of the nanoparticles [75,77].

**Figure 7:**
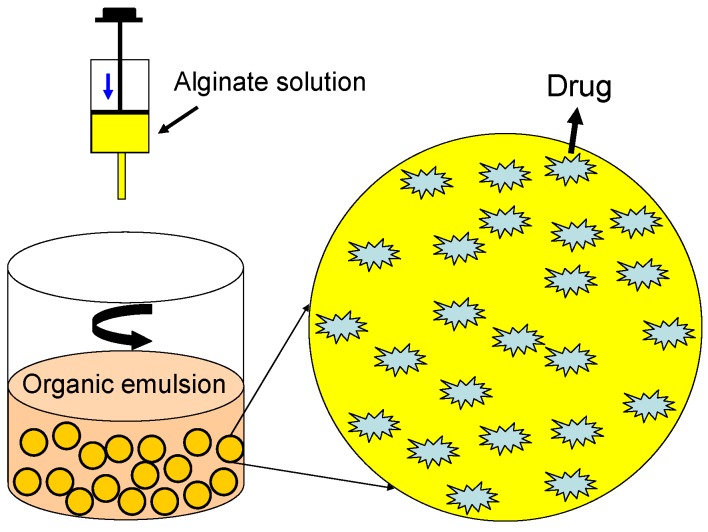
Illustration of the procedure for alginate solid-spheres in loading drugs.

Alginate microspheres and nanoparticles showing hydrophilic properties and an electronegative surface are necessary to avoid their uptake [86,87]. Technically, drugs can be loaded in alginate microspheres by using an emulsion solvent technique. Drugs can be mixed with the alginate solution evenly, and the mixture should then be emulsified under sonication. Drug-loaded alginate microspheres can be fabricated by adding the mixture dropwise to an organic emulsion with constant stirring (Figure 7). The alginate-based carriers can protect drugs from degradation and may improve plasma half time to ensure transport and release of drugs. In addition to carrying drugs, the alginate-based solid-microspheres also can be employed as cell microcarriers, another kind of injectable cell scaffold for tissue engineering.

### 2.3. Porous Scaffolds 

Currently, many porous scaffolds with highly functional properties have been utilized in the field of tissue engineering [88,89]. They can be applied as delivery vehicles for bioactive molecules, and as three-dimensional structures that organize cells, serving as a temporary skeleton to accommodate and stimulate new tissue growth [90,91]. Alginate can be easily formulated into porous scaffolding matrices of various forms (spheres, sponges, foams, fibers and rods) for cell culture and response, which makes it particularly suitable for regenerative medicine applications.

#### 2.3.1. Freeze-Dried Scaffolds

**Figure 8:**
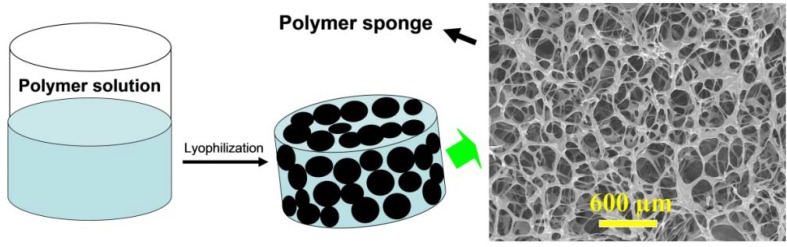
Schematic illustration to show the fabricating procedures of alginate-based sponge by the freeze-drying method.

Traditional methods for producing porous biopolymer scaffolds include gas foaming, freeze-drying, solvent casting, phase separation and particulate leaching. Compared to the others, freeze-drying is the easiest method to fabricate porous scaffolds [92,93,94]. Porous alginate-based scaffolds or sponges with interconnected porous structures and predictable shapes can be easily manufactured by a simple freeze-drying step (Figure 8). The mechanical properties and biodegradation rate of freeze-dried scaffolds can be simply modulated by changing the relative parameters of the polymers [95,96,97,98]. The mechanical strength mainly depends on porous scaffold forms and structural parameters such as pore size, porosity, and orientation. However, the diameter of the pores in freeze-dried scaffolds may not be uniform. The material components and molecular weight can strongly affect the biodegradation rates of scaffolds.

Porous scaffolds formed by pure alginate are unable to provide enough bioactive properties to support cell metabolism due to lack of cellular interaction in the molecular structures [97,98,99]. Therefore, alginate has been blended with collagen or gelatin to enhance cell ligand-specific binding properties to fabricate hybrid scaffolds, which showed better properties for supporting cells [42,81,83,98,99]. In a recent report, other efforts were made to enhance the biological properties of alginate porous scaffolds. For example, alginate was irradiated and oxidized to modify its degradation, and covalently grafted with growth factors, lectins and peptides containing a RGD sequence to promote cell adhesion and proliferation [100].

#### 2.3.2. Electrospun Nanofibers

**Figure 9:**
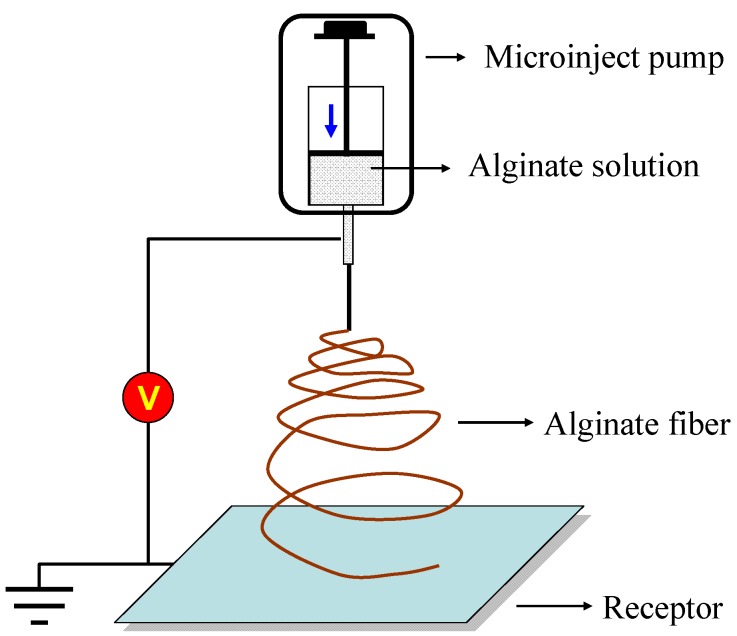
Illustration of electrospinning of an alginate fibrous scaffold.

For tissue regeneration applications, one role of cell scaffolds is to mimic ECM and provide structural support for developing tissues. Ideal cell scaffolds should be analogous to native ECM in terms of both chemical composition and physical structure. An alginate-based nanofiber matrix is similar, with its nanoscaled nonwoven fibrous ECM proteins, and thus is a candidate ECM-mimetic material [101,102]. Electrospinning is a facile method to fabricate alginate nanofibrous mats, which have a range of applications extending far beyond regenerative medicine (Figure 9). The feature sizes of electrospun mats, such as fiber diameters, can be tailored by the solution properties (e.g., viscosity, concentration) and process conditions (e.g., flowrate, electric field). The mat thickness is also affected by the total mass of deposited fibers and size of the collector plate.

Although alginate-based electrospun mats have shown promise as tissue scaffolds, their feature sizes and topography also have drawbacks. Specifically, electrospun nanofiber mats have a relatively flat topography, limited thickness, and dense fiber packing; as such, when used as tissue scaffolds, cell infiltration is restricted to the top layers of the electrospun mat [102]. Hence, a traditional electrospun nanofiber mat without modification may have limited use in regenerative medicine. For tissue engineering applications, electrospun alginate mat formations have been tailored by a variety of approaches in order to expand their capabilities [101,102].

## 3. Applications

The need for alginate-based biomaterials in tissue engineering and drug delivery is immense. In particular, as stem cells play an increasingly prominent role in the field of regenerative medicine [17,18], the combination and interaction between stem cells and alginate-based materials have been specifically emphasized. Analyzed by *in vitro* cytotoxicity assay and *in vitro* implantation, alginate-based microcapsules and scaffolds have shown minimal or negligible cytotoxicity and are histocompatible [103,104,105]. These *in vitro* results suggested tunable interactions between the multiple platelet releasate-derived bioagents and the biocomposites for enhancing hematoma-like fracture repair. Additionally, minimally invasive delivery for *in situ* curing of the implant systems via injection was demonstrated in rat tail vertebrae using microcomputed tomography. These results demonstrated that alginate-based scaffolds were able to degrade, allowed vascularization and elicited low inflammatory responses after transplantation. Therefore, alginate-based scaffolds can provide appropriate properties as potential cell and drug carriers for tissue regeneration. The following sections describe the pre-clinical and clinical studies of alginate-based biomaterials for these applications.

### 3.1. Wound Healing

Dressing has been applied to open wounds for centuries [106]. It can prevent wounds from further injury and bacteria invasion. Gauze is the simplest and most widely used dressing, having many advantages such as easy handling, great absorbent capability, and low cost. However, gauze may easily create secondary injury when peeling off. Nowadays, high quality wound dressings are designed to create a moist occlusive environment to promote healing. Many kinds of dressings such as sponge, gel, occlusive or semi-occlusive dressings have been reported.

Alginate has been used in a number of wound dressings. Alginate-based wound dressings such as sponges, hydrogels and electrospun mats are promising substrates for wound healing that offer many advantages including hemostatic capability and gel-forming ability upon absorption of wound exudates [107,108]. Alginate was found to possess many critical elements desirable in a wound dressing such as good water absorptivity, conformability, optimal water vapor transmission rate, and mild antiseptic properties coupled with nontoxicity and biodegradability. It has been suggested that certain alginate dressings (e.g., Kaltostat®) can enhance wound healing by stimulating monocytes to produce elevated levels of cytokines such as interleukin-6 and tumor necrosis factor-α [60]. Production of these cytokines at wound sites results in pro-inflammatory factors that are advantageous to wound healing. The high level of bioactivity of these dressings is believed to be due to the presence of endotoxin in alginates. Balakrishnan *et al.* showed that an *in situ*-forming hydrogel wound dressing can be prepared from gelatin and oxidized alginate in the presence of small concentrations of borax [60]. The composite matrix has the hemostatic effect of gelatin, the wound-healing promoting feature of alginate and the antiseptic property of borax to make it a potential wound dressing material.

Additionally, since the structure lacks signal sequence for cell adhesion, alginate-based dressings are popular for wound management and can avoid secondary injury when peeling off. Especially, wound dressings of alginate-based sponge are commonly used to treat the wound with large volume exudation. As a moist wound environment has been known to promote healing, wound dressings of alginate-based gel can prevent the wound bed from drying out, which leads to a better cosmetic repair of the wounds. Antimicrobial properties of wound dressings play a key role in determining the process of wound repair because wounds often provide favorable environments for colonization of microorganisms, which may lead to infection and delay healing. Alginate was combined with chitosan and Ag nano-particles to form an antibacterial wound dressing. Based on the advantages of alginate and water-soluble chitosan, a composite polysaccharide sponge was fabricated, resulting in an anti-adhesive and antimicrobial wound dressing (Figure 8).

### 3.2. Cartilage Repair

The need for tissue-engineered cartilage is immense and of great clinical significance. Traumatic and degenerative lesions of articular cartilage are leading causes of disability [109,110,111]. It is estimated that over 100 million Chinese currently suffer from osteoarthritis. Tissue engineering methods to improve cartilage repair and regeneration will therefore have high clinical impact. The advantage of injectable therapies for cartilage repair is that the implant is not only maintained within the defect, but also allows immediate weight-bearing due to the stiffness and strength that is achieved almost instantly [112,113,114]. The physical properties of the alginate hydrogel can be designed to easily match those of articular cartilage in addition to matching the mechanical properties of the scaffold with the native tissue. Alginate-based injectable hydrogels, solid- and gel-microspheres have been used in cartilage regeneration.

Many researchers have studied the combination of alginate-based microspheres and hydrogels for controlled growth factor delivery in tissue engineering [115,116,117,118,119,120,121,122]. For example, a study demonstrated the positive effect of immobilizing RGD to a macro-porous alginate scaffold in promoting TGF-*β*-induced human MSC differentiation [121]. The cell-matrix interactions facilitated by the immobilized RGD peptide were shown to be an essential feature of the cell microenvironment, allowing better cell accessibility to the chondrogenic-inducing molecule TGF-*β*. Bian *et al.* investigated the co-encapsulation of TGF-*β* containing alginate microspheres with human MSCs in hyaluronic acid (HA) hydrogels with regard to the development of implantable constructs for cartilage repair [86]. TGF-*β* loaded alginate microspheres combined with hydrogels form a composite carrier which may help to retain TGF-*β* bioactivity in the scaffold and promote chondrogenesis of MSCs when implanted. Wang *et al.* prepared an organized 3D alginate microsphere scaffold using a microfluidic device, which was effective for chondrocyte culture *in vitro* [122]. The animal experiment showed that chondrocytes seeded into the alginate microsphere scaffold survived normally in SCID mice, and cartilage-like structures were formed after four weeks implantation.

### 3.3. Bone Regeneration

Bone regeneration is a significant challenge in reconstructive surgery. There are several reasons for lack of bone tissue, such as trauma and tumor removal. A desirable strategy to repair bone tissue is to induce osteogenesis *in situ*. One method to accomplish this is to utilize stem cells that can differentiate to form bone tissue, and seed those cells into an injectable scaffold, resulting in bone tissue formation [123,124,125,126,127,128]. As such, there have been numerous studies involving the use of injectable alginate-based scaffolds for bone regeneration [128,129,130,131,132,133,134,135]. Adequate bone tissue formation was observed using MSCs and alginate as the scaffold [130,131,132,133]. Alginate, therefore, is applicable for generating tissue in gels, displaying osteogenic as well as angiogenic properties.

Many researchers reported bone regeneration using injectable scaffolds combining alginate-based hydrogels or microspheres which were mixed with undifferentiated MSCs or ASCs [127,128,129,130,131,132,133,134]. These studies demonstrated the potential of bone morphogenetic protein (BMP) and TGF-*β* delivery to induce osteogenic differentiation to mature osteocytes from MSCs and ASCs [129,130,131,132]. Kolambkar *et al.* introduced a hybrid growth factor delivery system that consists of an electrospun nanofiber mesh tube for guiding bone regeneration combined with a peptide-modified alginate hydrogel injected inside the tube for sustained recombinant BMP-2 (rhBMP-2) release [134]. The results indicated that sustained delivery of rhBMP-2 via alginate hydrogel was required for substantial regeneration to occur. This hybrid technique may be clinically useful for bone regeneration in the case of fracture of non-unions and large bone defects.

Present findings showed that the co-immobilization of osteogenic and endothelial cells within RGD-alginate microspheres is a promising new injectable strategy for bone tissue engineering [126]. Endothelial cells could regulate the osteogenic potential of osteoprogenitor cells *in vivo* and *in vitro* when co-immobilized within alginate microspheres modified with the RGD sequence. *In vitro* three-dimensional dynamic studies showed increased cell metabolic activity and upregulation of gene expression of alkaline phosphatase and osteocalcin, as well as mineralization, when osteoprogenitor cells were co-immobilized with endothelial cells. After implantation in a long bone defect, *in vivo* studies showed that immobilized cells promoted mineralization of the microspheres, which was significantly enhanced when osteoprogenitors were co-immobilized with endothelial cells.

### 3.4. Drug Delivery

Drug-delivery carriers have attracted a lot of interest during the past decades, since they can deliver low-molecular-weight drugs, as well as large biomacromolecules such as proteins and genes, either in a localized or in a targeted manner [136,137]. Alginate has been widely adopted as a carrier to immobilize or encapsulate drugs, bioactive molecules, proteins and cells, for its biocompatible and biodegradable nature [137,138,139,140]. To date, many types of alginate-based carriers, such as hydrogels, colloidal particles, and polyelectrolyte complexes, are under investigation, and some of them have been used practically. A number of researchers have studied the combination of alginate-based hydrogels, porous scaffolds and microspheres for controlled drug delivery in tissue engineering [138,139].

Alginate-based hollow microcapsules have great potential application as drug-delivery vehicle, biosensors and micro-reactors [140]. Hollow microcapsules can be constructed by means of sequential self-assembly of negatively and positively charged polyelectrolytes, namely the layer-by-layer (LbL) technique. Acting as drug-delivery carriers, the microcapsules have been well studied with respect to controllable loading and release properties. Attempts have also been made to fabricate biopolymer microcapsules by depositing chitosan/alginate onto decomposable colloid particles, followed by core removal with suitable pathways. For example, alginate and chitosan were alternately deposited onto CaCO_3_ particles to produce hollow microcapsules with expectable biocompatibility to electrostatic interaction [140]. The properties and functionalities of alginate/chitosan microcapsules can be fine-tuned by varying the microcapsule wall thickness, composition and the introduction of exterior stimuli. Degradation studies have been carried out by immersing the microcapsules in solutions of different pH values to investigate the role of the material as well as the number of encapsulation layers in maintaining the stability of the microcapsules in the different pH environments. Wong’s study revealed that the addition of PEG to the alginate-based microcapsules led to protection against an acidic environment, whilst the number of coating layers only influences the swelling properties and not the degradation and Young’s modulus of the microcapsules [141].

Recently, the use of a tissue engineering approach for developing a 3D high throughput screening assay for drug screening and diagnostic devices has been of great interest [142,143,144,145]. An alginate hydrogel has been utilized as a 3D platform for microarray systems as well as surface micro-patternings. Small aliquots of a gelation solution were selectively trapped on the hydrophilic areas by a simple dipping process, utilized to make thin hydrogel patterns by *in situ* gelation. The alginate gel-patterns were used to capture cells with different adhesion properties selectively on or off the hydrogel structures. The up-regulation of several CYP450 enzymes, *β*1-integrin and vascular endothelial growth factor (VEGF) in the 3D microarray cultures suggested that the platform provided a more *in vitro*-like environment allowing cells to approach their natural phenotypes.

For modulating bioactivity signals, gene delivery has gained increasing interest in tissue repair and regeneration [146]. Plasmid DNAs are expected to transfect cells *in situ* and express the required growth factors. Small interfering RNAs (siRNAs) are used to silence targeted genes and down-regulate the corresponding protein levels. Although alginates have been independently applied in drug-delivery carrier fabrication, alginate-based delivery systems for functional DNAs or siRNAs are more promising but have been hardly reported up to now. In this type of application, modified alginate with cationic properties is necessary to enhance the transfection efficiency of DNAs or siRNAs to target cells.

## 4. Summary

To summarize, alginate has been extensively utilized in biomaterials or in building blocks for tissue repair and regeneration. Physical and chemical modifications are carried out to derive alginates with the desired structures, properties, and functions. Alginate-based biomaterials are promising substrates for tissue engineering with the advantage that both drugs and cells can be readily integrated into the scaffolding matrix. The success of tissue constructs is highly dependent on the design of the alginate-based scaffolds including the physical, chemical and biological properties. Successful exploitation of alginate-based biomaterials in different tissues and organs such as skin, cartilage, and bone suggests their promising future for repair and regeneration applications. However, current alginate is still unable to meet all the design parameters simultaneously (e.g., degradation, bioactivities or mechanical properties). In further studies, efforts should be made to improve alginate and thus, support the development of more natural and functional tissues. Cell induction ligands such as growth factors can be incorporated into alginate-based scaffolds such that specific signals can be delivered in an appropriate spatial and temporal manner. More alginate-based biomaterials occupying novel physical, chemical and biological properties should be developed to mimic the environment of natural tissues. Smart hydrogels and porous scaffolds are important applicable material forms, while the alginate-based delivery systems for bioactive signaling molecules, functional DNAs or siRNAs are also of great significance in constructing bioactive biomaterials.

## References

[1] Lee K.Y., Mooney D.J. (2012). Alginate: Properties and biomedical applications. Prog. Polym. Sci..

[2] Lee K.Y., Yuk S.H. (2007). Polymeric protein delivery systems. Progr. Polym. Sci..

[3] Pawar S.N., Edgar K.J. (2012). Alginate derivatization: a review of chemistry, properties and applications. Biomaterials.

[4] Tan H., Gong Y., Lao L., Mao Z., Gao C. (2007). Gelatin/chitosan/hyaluronan ternary complex scaffold containing basic fibroblast growth factor for cartilage tissue engineering. J. Mater. Sci. Mater. Med..

[5] Senni K., Pereira J., Gueniche F., Delbarre-Ladrat C., Sinquin C., Ratiskol J., Godeau G., Fischer A.M., Helley D., Colliec-Jouault S. (2011). Marine polysaccharides: A source of bioactive molecules for cell therapy and tissue engineering. Mar. Drugs.

[6] Wu J., Tan H., Li L., Gao C. (2009). Covalently immobilized gelatin gradients within three-dimensional porous scaffolds. Chin. Sci. Bull..

[7] Narayanan R.P., Melman G., Letourneau N.J., Mendelson N.L., Melman A. (2012). Photodegradable iron(III) cross-linked alginate gels. Biomacromolecules.

[8] Skjak-Braerk G., Grasdalen H., Smidsrod O. (1989). Inhomogeneous polysaccharide ionic gels. Carbohydr. Polym..

[9] Stevens M.M., Qanadilo H.F., Langer R., Shastri V.P. (2004). A rapid-curing alginate gel system: Utility in periosteum-derived cartilage tissue engineering. Biomaterials.

[10] Kuo C.K., Ma P.X. (2001). Ionically crosslinked alginate hydrogels as scaffolds for tissue engineering: Part 1. Structure, gelation rate and mechanical properties. Biomaterials.

[11] Bouhadir K.H., Lee K.Y., Alsberg E., Damm K.L., Anderson K.W., Mooney D.J. (2001). Degradation of partially oxidized alginate and its potential application for tissue engineering. Biotechnol. Prog..

[12] Kong H.J., Alsberg E., Kaigler D., Lee K.Y., Mooney D.J. (2004). Controlling degradation of hydrogel via the size of cross-linked junctions. Adv. Mater..

[13] Balakrishnan B., Jayakrishnan A. (2005). Self-cross-linking biopolymers as injectable *in situ* forming biodegradable scaffolds. Biomaterials.

[14] Gaserod O., Smidsrod O., Skjak-Braek G. (1998). Microcapsules of alginate-chitosan I: A quantitative study of the interaction between alginate and chitosan. Biomaterials.

[15] Rowley J.A., Madlambayan G., Mooney D.J. (1999). Alginate hydrogels as synthetic extracellular matrix materials. Biomaterials.

[16] Lee K.Y., Mooney D.J. (2001). Hydrogels for tissue engineering. Chem. Rev..

[17] Tan H., Marra K.G. (2010). Injectable, biodegradable hydrogels for tissue engineering applications. Materials.

[18] Tememoff J.S., Mikos A.G. (2000). Injectable biodegradable materials for orthopedic tissue engineering. Biomaterials.

[19] Hou Q.P., de Bank P.A., Shakesheff K.M. (2004). Injectable scaffolds for tissue regeneration. J. Mater. Chem..

[20] Drury J.L., Mooney D.J. (2003). Hydrogels for tissue engineering: scaffold design variables and applications. Biomaterials.

[21] Nuttelman C.R., Rice M.A., Rydholm A.E., Salinas C.N., Shah D.N., Anseth K.S. (2008). Macromolecular monomers for the synthesis of hydrogel niches and their application in cell encapsulation and tissue engineering. Prog. Polym. Sci..

[22] Brandl F., Sommer F., Goepferich A. (2007). Rational design of hydrogels for tissue engineering: Impact of physical factors on cell behavior. Biomaterials.

[23] Rehfeldt R., Engler A.J., Eckhardt A., Ahmed F., Discher D.E. (2007). Cell responses to the mechanochemical microenvironment—Implications for regenerative medicine and drug delivery. Adv. Drug Deliv. Rev..

[24] Nicodemus G.D., Bryant S.J. (2008). Cell encapsulation in biodegradable hydrogels for tissue Engineering applications. Tissue Eng..

[25] Varghese S., Elisseeff J.H. (2006). Hydrogels for musculoskeletal tissue engineering. Adv. Polym. Sci..

[26] Tan H., DeFail A.J., Rubin J.P., Chu C.R., Marra K.G. (2009). Novel multi-arm PEG-based hydrogels for tissue engineering. J. Biomed. Mater. Res. A.

[27] Tan H., Xiao C., Sun J., Xiong D., Hu X. (2012). Biological self-assembly of injectable hydrogel as cell scaffold via specific nucleobase pairing. Chem. Commun..

[28] Tan H., Rubin J.P., Marra K.G. (2011). Direct synthesis of biodegradable polysaccharide derivative hydrogels through aqueous Diels-Alder chemistry. Macromol. Rapid Commun..

[29] Donati I., Holtan S., Mørch Y.A., Borgogna M., Dentini M., Skjåk-Bræk G. (2005). New hypothesis on the role of alternating sequences in calcium-alginate gels. Biomacromolecules.

[30] Crow B.B., Nelson K.D. (2006). Release of bovine serum albumin from a hydrogel-cored biodegradable polymer fiber. Biopolymers.

[31] Ruvinov E., Leor J., Cohen S. (2010). The effects of controlled HGF delivery from an affinity-binding alginate biomaterial on angiogenesis and blood perfusion in a hind limb ischemia model. Biomaterials.

[32] Gan T., Zhang Y., Guan Y. (2009). *In situ* gelation of P(NIPAM-HEMA) microgel dispersion and its applications as injectable 3D cell scaffold. Biomacromolecules.

[33] Kim J.H., Lee S.S., Kim S.J., Lee Y.M. (2002). Rapid temperature/pH response of porous alginate-g-poly(*N*-isopropylacrylamide) hydrogels. Polymer.

[34] Lee S.B., Ha D.I., Cho S.K., Kim S.J., Lee Y.M. (2004). Temperature/pH-sensitive comb-type graft hydrogels composed of chitosan and poly(*N*-isopropylacrylamide). J. Appl. Polym. Sci..

[35] Lee J.W., Jung M.C., Park H.D., Park K.D., Ryu G.H. (2004). Synthesis and characterization of thermosensitive chitosan copolymer as a novel biomaterial. J. Biomed. Mater. Res..

[36] Wang J., Chen L., Zhao Y., Guo G., Zhang R. (2009). Cell adhesion and accelerated detachment on the surface of temperature-sensitive chitosan and poly(*N*-isopropylacrylamide) hydrogels. J. Mater. Sci. Mater. Med..

[37] Chen J.P., Cheng T.H. (2006). Thermo-responsive chitosan-graft-poly(*N*-isopropylacrylamide) injectable hydrogel for cultivation of chondrocytes and meniscus cells. Macromol. Biosci..

[38] Cho J.H., Kim S.H., Park K.D., Jung M.C., Yang W.I., Han S.W., Noh J.Y., Jin J.W., Lee W. (2004). Chondrogenic differentiation of human mesenchymal stem cells using a thermosensitive poly(*N*-isopropylacrylamide) and water-soluble chitosan copolymer. Biomaterials.

[39] Ha D.I., Lee S.B., Chong M.S., Lee Y.M., Kim S.Y., Park Y.H. (2006). Preparation of thermo-responsive and injectable hydrogels based on hyaluronic acid and poly(*N*-isopropylacrylamide) and their drug release behaviors. Macromol. Res..

[40] Ibusuki S., Fujii Y., Iwamoto Y., Matsuda T. (2003). Tissue-engineered cartilage using an injectable and *in situ* gelable thermoresponsive gelatin: fabrication and *in vitro* performance. Tissue Eng..

[41] Tan H., Ramirez C.M., Miljkovic N., Li H., Rubin J.P., Marra K.G. (2009). Thermosensitive injectable hyaluronic acid hydrogel for adipose tissue engineering. Biomaterials.

[42] Abdi S.I.H., Choi J.Y., Lee J.S., Lim H.J., Lee C., Kim J., Chung H.Y., Lim J.O. (2012). *In vitro* study of a blended hydrogel composed of Pluronic F-127-alginate-hyaluronic acid for its cell injection application. J. Tissue Eng. Regen. Med..

[43] Lee K.Y., Kong H.J., Larson R.G., Mooney D.J. (2003). Hydrogel formation *via* cell crosslinking. Adv. Mater..

[44] Lehenkari P.P., Horton M.A. (1999). Single integrin molecule adhesion forces in intact cells measured by atomic force microscopy. Biochem. Bio-phys. Res. Commun..

[45] Koo L.Y., Irvine D.J., Mayes A.M., Lauffenburger D.A., Griffith L.G. (2002). Coregulation of cell adhesion by nanoscale RGD organization and mechanical stimulus. J. Cell Sci..

[46] Schmedlen R.H., Masters K.S., West J.L. (2002). Photocrosslinkable polyvinyl alcohol hydrogels that can be modified with cell adhesion peptides for use in tissue engineering. Biomaterials.

[47] Hu X., Gao C. (2008). Photoinitiating polymerization to prepare biocompatible chitosan hydrogels. J. Appl. Polym. Sci..

[48] Ifkovits J.L., Burdick J.A. (2007). Review: photopolymerizable and degradable biomaterials for tissue engineering applications. Tissue Eng..

[49] Varghese S., Hwang N.S., Canver A.C., Theprungsirikul P., Lin D.W., Elisseeff J. (2008). Chondroitin sulfate based niches for chondrogenic differentiation of mesenchymal stem cells. Matrix Biology.

[50] Park Y.D., Tirelli N., Hubbell J.A. (2003). Photopolymerized hyaluronic acid-based hydrogels and interpenetrating networks. Biomaterials.

[51] DeLong S.A., Gobin A.S., West J.L. (2005). Covalent immobilization of RGDS on hydrogel surfaces to direct cell alignment and migration. J. Control. Rel..

[52] Garagorri N., Fermanian S., Thibault R., Ambrose W.M., Schein O.D., Chakravarti S., Elisseeff J. (2008). Keratocyte behavior in three-dimensional photopolymerizable poly(ethylene glycol) hydrogels. Acta Biomater..

[53] Bryant S.J., Anseth K.S., Lee D.A., Bader D.L. (2004). Crosslinking density influences the morphology of chondrocytes photoencapsulated in PEG hydrogels during the application of compressive strain. J. Orthop. Res..

[54] Rice M.A., Anseth K.S. (2004). Encapsulating chondrocytes in copolymer gels: Bimodal degradation kinetics influence cell phenotype and extracellular matrix development. J. Biomed. Mater. Res..

[55] Bryant S.J., Bender R., Durand K.L., Anseth K.S. (2004). Encapsulating chondrocytes in degrading PEG hydrogels with high modulus: Engineering gel structural changes to facilitate cartilaginous tissue production. Biotechnol. Bioeng..

[56] Peter S.J., Lu L.C., Mikos A.G. (2000). Marrow stormal osteoblast function on a poly(propylene fumarate)/β-tricalcium phosphate biodegradable orthopaedic composite. Biomaterials.

[57] He S.L., Yaszemski M.J., Yasko A.W., Engel P.S., Mikos A.G. (2000). Injectable biodegradable polymer composites based on poly(propylene fumarate) crosslinked with poly(ethylene glycol)-dimethacrylate. Biomaterials.

[58] Temenoff J.S., Park H., Jabbari E., Sheffield T.L., LeBaron R.G., Ambrose C.G., Mikos A.G. (2004). *In vitro* osetogenic differentiation of marrow stromal cells encapsulated in biodegradable hydrogels. J. Biomed. Mater. Res..

[59] Cha C., Kim E.S., Kim I.W., Kong H. (2011). Integrative design of a poly(ethylene glycol)-poly(propylene glycol)-alginate hydrogel to control three dimensional biomineralization. Biomaterials.

[60] Balakrishnan B., Mohanty M., Umashankar P.R., Jayakrishnan A. (2005). Evaluation of an *in situ* forming hydrogel wound dressing based on oxidized alginate and gelatin. Biomaterials.

[61] Dahlmann J., Krause A., Möller L., Kensah G., Möwes M., Diekmann A., Martin U., Kirschning A., Gruh I., Dräger G. (2013). Fully defined *in situ* cross-linkable alginate and hyaluronic acid hydrogels for myocardial tissue engineering. Biomaterials.

[62] Boontheekul T., Kong H., Mooney D. (2005). Controlling alginate gels degradation utilizing partial oxidation and bimodal molecular weight distribution. Biomaterials.

[63] Tan H., Chu C.R., Payne K.A., Marra K.G. (2009). Injectable *in situ* forming biodegradable chitosan-hyaluronic acid based hydrogels for cartilage tissue engineering. Biomaterials.

[64] Krause A., Kirschning A., Dräger G. (2012). Bioorthogonal metal-free click-ligation of cRGD-pentapeptide to alginate. Org. Biomol. Chem..

[65] Tan H., Hu X. (2012). Injectable *in situ* forming glucose-responsive dextran-based hydrogels to deliver adipogenic factor for adipose tissue engineering. J. Appl. Polym. Sci..

[66] Tan H., Li H., Rubin J.P., Marra K.G. (2011). Controlled gelation and degradation rates of injectable hyaluronic acid-based hydrogels through a double crosslinking strategy. J. Tissue Eng. Regen. Med..

[67] Tan H., Rubin J.P., Marra K.G. (2010). Injectable *in situ* forming biodegradable chitosan-hyaluronic acid based hydrogels for adipose tissue regeneration. Organogenesis.

[68] Springer M.L., Hortelano G., Bouley D.M., Wong J., Kraft P.E., Blau H.M. (2000). Induction of angiogenesis by implantation of encapsulated primary myoblasts expressing vascular endothelial growth factor. J. Gene Med..

[69] Tan H., Huang D., Lao L., Gao C. (2009). RGD modified PLGA/gelatin microspheres as microcarriers for chondrocyte delivery. J. Biomed. Mater. Res..

[70] Patil S.B., Sawant K.K. (2008). Mucoadhesive microspheres: a promising tool in drug delivery. Curr. Drug Deliv..

[71] Basmanav B.F., Kose G.T., Hasirci V. (2008). Sequential growth factor delivery from complexed microspheres for bone tissue engineering. Biomaterials.

[72] Chang T.M. (1998). Pharmaceutical and therapeutic applications of artificial cells including microencapsulation. Eur. J. Pharm. Biopharm..

[73] Serra M., Correia C., Malpique R., Brito C., Jensen J., Bjorquist P., Carrondo M.J., Alves P.M. (2011). Microencapsulation technology: A powerful tool for integrating expansion and cryopreservation of human embryonic stem cells. PLoS One.

[74] Kong H.J., Smith M.K., Mooney D.J. (2003). Designing alginate hydrogels to maintain viability of immobilized cells. Biomaterials.

[75] Man Y., Wang P., Guo Y., Xiang L., Yang Y., Qu Y., Gong P., Deng L. (2012). Angiogenic and osteogenic potential of platelet-rich plasma and adipose-derived stem cell laden alginate microspheres. Biomaterials.

[76] Yu J., Du K.T., Fang Q. (2010). The use of human mesenchymal stem cells encapsulated in RGD modified alginate microspheres in the repair of myocardial infarction in the rat. Biomaterials.

[77] Freiberg S., Zhu X.X. (2004). Polymer microspheres for controlled drug release. Int. J. Pharm..

[78] Chen L., Subirade M. (2006). Alginate—Whey protein granular microspheres as oral delivery vehicles for bioactive compounds. Biomaterials.

[79] Liu J., Zhou H., Weir M.D., Xu H., Chen Q., Trotman C.A. (2012). Fast-degradable microbeads encapsulating human umbilical cord stem cells in alginate for muscle tissue engineering. Tissue Eng. Part A.

[80] Huang X., Zhang X., Wang X., Wang C., Tang B. (2012). Microenvironment of alginate-based microcapsules for cell culture and tissue engineering. J. Biosci. Bioeng..

[81] Yao R., Zhang R., Luan J., Lin F. (2012). Alginate and alginate/gelatin microspheres for human adipose-derived stem cell encapsulation and differentiation. Biofabrication.

[82] Olderøy M.O., Xie M., Andreassen J.P., Strand B.L., Zhang Z., Sikorski P. (2012). Viscoelastic properties of mineralized alginate hydrogel beads. J. Mater. Sci. Mater. Med..

[83] Zheng H., Tian W., Yan H., Yue L., Zhang Y., Han F., Chen X., Li Y. (2012). Rotary culture promotes the proliferation of MCF-7 cells encapsulated in three-dimensional collagen-alginate hydrogels via activation of the ERK1/2-MAPK pathway. Biomed. Mater..

[84] Wang C., Yang K., Lin K., Liu H., Lin F. (2011). A highly organized three-dimensional alginate scaffold for cartilage tissue engineering prepared by microfluidic technology. Biomaterials.

[85] Tan H., Wu J., Huang D., Gao C. (2010). The design of biodegradable microcarriers for induced cell aggregation. Macromol. Biosci..

[86] Bian L., Zhai D.Y., Tous E., Rai R., Mauck R.L., Burdick J.A. (2011). Enhanced MSC chondrogenesis following delivery of TGF-*β*3 from alginate microspheres within hyaluronic acid hydrogels *in vitro* and *in vitro*. Biomaterials.

[87] Soran Z., Aydın R.S., Gümüşderelioğlu M. (2012). Chitosan scaffolds with BMP-6 loaded alginate microspheres for periodontal tissue engineering. J. Microencapsul..

[88] Tan H., Wu J., Lao L., Gao C. (2009). Gelatin/chitosan/hyaluronan scaffold integrated with PLGA microspheres for cartilage tissue engineering. Acta Biomater..

[89] Tan H., Zhou Q., Qi H., Zhu D., Ma X., Xiong D. (2012). Heparin interacting protein mediated assembly of nano-fibrous hydrogel scaffolds for guided stem cell differentiation. Macromol. Biosci..

[90] Petite H., Viateau V., Bensaid W., Meunier A., de Pollak C., Bourguignon M., Oudina K., Sedel L., Guillemin G. (2000). Tissue-engineered bone regeneration. Nat. Biotechnol..

[91] Kuo Y.C., Chung C.Y. (2012). TATVHL peptide-grafted alginate/poly(γ-glutamic acid) scaffolds with inverted colloidal crystal topology for neuronal differentiation of iPS cells. Biomaterials.

[92] Yang S., Leong K.F., Du Z., Chua C. (2001). The design of scaffolds for use in tissue engineering. Part I: Traditional factors. Tissue Eng..

[93] Becker T.A., Kipke D.R., Brandon T. (2001). Calcium alginate gel: A biocompatible and mechanically stable polymer for endovascular embolization. J. Biomed. Mater. Res..

[94] Wu X., Liu Y., Li X., Wen P., Zhang Y., Long Y., Wang X., Guo Y., Xing F., Gao J. (2010). Preparation of aligned porous gelatin scaffolds by unidirectional freeze-drying method. Acta Biomater..

[95] Bhardwaj N., Kundu S.C. (2010). Electrospinning: A fascinating fiber fabrication technique. Biotechnol. Adv..

[96] George P.A., Quinn K., Cooper-White J.J. (2010). Hierarchical scaffolds via combined macro- and micro-phase separation. Biomaterials.

[97] Salerno A., Oliviero M., Maio D.E., Iannace S., Netti P.A. (2009). Design of porous polymeric scaffolds by gas foaming of heterogeneous blends. J. Mater. Sci. Mater. Med..

[98] Sapir Y., Kryukov O., Cohen S. (2011). Integration of multiple cell-matrix interactions into alginate scaffolds for promoting cardiac tissue regeneration. Biomaterials.

[99] Florczyk S.J., Kim D.J., Wood D.L., Zhang M. (2011). Influence of processing parameters on pore structure of 3D porous chitosan-alginate polyelectrolyte complex scaffolds. J. Biomed. Mater. Res. A.

[100] Shachar M., Tsur-Gang O., Dvir T., Leor J., Cohen S. (2011). The effect of immobilized RGD peptide in alginate scaffolds on cardiac tissue engineering. Acta. Biomater..

[101] Kang E., Choi Y.Y., Chae S.K., Moon J.H., Chang J.Y., Lee S.H. (2012). Microfluidic spinning of flat alginate fibers with grooves for cell-aligning scaffolds. Adv. Mater..

[102] Bonino C.A., Efimenko K., Jeong S.I., Krebs M.D., Alsberg E., Khan S.A. (2012). Three-dimensional electrospun alginate nanofiber mats via tailored charge repulsions. Small.

[103] McCanless J.D., Jennings L.K., Bumgardner J.D., Cole J.A., Haggard W.O. (2012). Hematoma-inspired alginate/platelet releasate/CaPO4 composite: initiation of the inflammatory-mediated response associated with fracture repair *in vitro* and *ex vivo* injection delivery. J. Mater. Sci. Mater. Med..

[104] De Vos P., Spasojevic M., de Haan B.J., Faas M.M. (2012). The association between *in vitro* physicochemical changes and inflammatory responses against alginate based microcapsules. Biomaterials.

[105] Vanacker J., Luyckx V., Dolmans M.M., Des Rieux A., Jaeger J., van Langendonckt A., Donnez J., Amorim C.A. (2012). Transplantation of an alginate-matrigel matrix containing isolated ovarian cells: first step in developing a biodegradable scaffold to transplant isolated preantral follicles and ovarian cells. Biomaterials.

[106] Xu H., Ma L., Shi H., Gao C., Han C. (2007). Chitosan-hyaluronic acid hybrid film as a novel wound dressing: *in vitro* and *in vitro* studies. Polym. Adv. Technol..

[107] Li X., Chen S., Zhang B., Li M., Diao K., Zhang Z., Li J., Xu Y., Wang X., Chen H. (2012). *In situ* injectable nano-composite hydrogel composed of curcumin, N,O-carboxymethyl chitosan and oxidized alginate for wound healing application. Int. J. Pharm..

[108] Hooper S.J., Percival S.L., Hill K.E., Thomas D.W., Hayes A.J., Williams D.W. (2012). The visualisation and speed of kill of wound isolates on a silver alginate dressing. Int. Wound J..

[109] Tan H., Wan L., Wu J., Gao C. (2008). Microscale control over collagen gradient on poly(L-lactide) membrane surface for manipulating chondrocyte distribution. Colloids Surf. B Biointerfaces.

[110] Tan H., Lao L., Wu J., Gong Y., Gao C. (2008). Biomimetic modification of chitosan with covalently grafted lactose and blended heparin for improvement of *in vitro* cellular interaction. Polym. Adv. Technol..

[111] Ferretti M., Marra K.G., Kobayashi K., DeFail A.J., Chu C.R. (2006). Controlled *in vitro* degradation of genipin crosslinked poly(ethylene glycol) hydrogels within osteochondral defects. Tissue Eng..

[112] Cooper C., Snow S., McAlindon T.E., Kellingray S., Stuart B., Coggon D. (2000). Risk factors for the incidence and progression of radiographic knee osteoarthritis. Arthritis Rheum..

[113] Awad H.A., Wickham M.Q., Leddy H.A., Gimble J.M., Guilak F. (2004). Chondrogenic differentiation of adipose-derived adult stem cells in agarose, alginate, and gelatin scaffolds. Biomaterials.

[114] Paige K.T., Cima L.G., Yaremchuk M.J., Schloo B.L., Vacanti J.P., Vacanti CA. (1996). De novo cartilage generation using calcium alginate-chondrocyte constructs. Plast. Reconstr. Surg..

[115] Lubiatowski P., Kruczynski J., Gradys A., Trzeciak T., Jaroszewski J. (2006). Articular cartilage repair by means of biodegradable scaffolds. Transplant Proc..

[116] Chen R., Curran S.J., Curran J.M., Hunt J.A. (2006). The use of poly(l-lactide) and RGD modified microspheres as cell carriers in a flow intermittency bioreactor for tissue engineering cartilage. Biomaterials.

[117] Henrionnet C., Wang Y., Roeder E., Gambier N., Galois L., Mainard D., Bensoussan D., Gillet P., Pinzano A. (2012). Effect of dynamic loading on MSCs chondrogenic differentiation in3-D alginate culture. Biomed. Mater. Eng..

[118] Ma K., Titan A.L., Stafford M., Zheng C., Levenston M.E. (2012). Variations in chondrogenesis of human bone marrow-derived mesenchymal stem cells in fibrin/alginate blended hydrogels. Acta Biomater..

[119] Coates E.E., Riggin C.N., Fisher J.P. (2012). Matrix molecule influence on chondrocyte phenotype and proteoglycan 4 expression by alginate-embedded zonal chondrocytes and mesenchymal stem cells. J. Orthop. Res..

[120] Ghahramanpoor M.K., Najafabadi S.A., Abdouss M., Bagheri F., Eslaminejad B.M. (2011). A hydrophobically-modified alginate gel system: utility in the repair of articular cartilage defects. J. Mater. Sci. Mater. Med..

[121] Reem T., Tsur-Gang O., Cohen S. (2010). The effect of immobilized RGD peptide in macroporous alginate scaffolds on TGFβ1-induced chondrogenesis of human mesenchymal stem cells. Biomaterials.

[122] Wang C., Yang K., Lin K., Liu Y., Liu H., Lin F. (2012). Cartilage regeneration in SCID mice using a highly organized three-dimensional alginate scaffold. Biomaterials.

[123] Alsberg E., Anderson K.W., Albeiruti A., Franceschi R.T., Mooney D.J. (2001). Cell-interactive alginate hydrogels for bone tissue engineering. J. Dent. Res..

[124] Abbah S.A., Lu W.W., Chan D., Cheung K.M., Liu W.G., Zhao F., Li Z.Y., Leong J.C.Y., Luk K.D.K. (2006). *In vitro* evaluation of alginate encapsulated adipose-tissue stromal cells for use as injectable bone graft substitute. Biochem. Biophys. Res. Commun..

[125] Durrieu M.C., Pallu S., Guillemot F., Bareille R., Amedee J., Baquey C.H., Labrugère C., Dard M. (2004). Grafting RGD containing peptides onto hydroxyapatite to promote osteoblastic cells adhesion. J. Mater. Sci. Mater. Med..

[126] Grellier M., Granja P.L., Fricain J.C., Bidarra S.J., Renard M., Bareille R., Bourget C., Amédée J., Barbosa M.A. (2009). The effect of the co-immobilization of human osteoprogenitors and endothelial cells within alginate microspheres on mineralization in a bone defect. Biomaterials.

[127] Jin H.H., Kim D.H., Kim T.W., Shin K.K., Jung J.S., Park H.C., Yoon S.Y. (2012). *In vitro* evaluation of porous hydroxyapatite/chitosan-alginate composite scaffolds for bone tissue engineering. Int. J. Biol. Macromol..

[128] Rubert M., Monjo M., Lyngstadaas S.P., Ramis J.M. (2012). Effect of alginate hydrogel containing polyproline-rich peptides on osteoblast differentiation. Biomed. Mater..

[129] Florczyk S.J., Leung M., Jana S., Li Z., Bhattarai N., Huang J., Hopper R.A., Zhang M. (2012). Enhanced bone tissue formation by alginate gel-assisted cell seeding in porous ceramic scaffolds and sustained release of growth factor. J. Biomed. Mater. Res. A.

[130] Tang M., Chen W., Weir M.D., Thein-Han W., Xu H. (2012). Human embryonic stem cell encapsulation in alginate microbeads in macroporous calcium phosphate cement for bone tissue engineering. Acta Biomater..

[131] Chen W., Zhou H., Weir M.D., Bao C., Xu H. (2012). Umbilical cord stem cells released from alginate-fibrin microbeads inside macroporous and biofunctionalized calcium phosphate cement for bone regeneration. Acta Biomater..

[132] Xia Y., Mei F., Duan Y., Gao Y., Xiong Z., Zhang T., Zhang H. (2012). Bone tissue engineering using bone marrow stromal cells and an injectable sodium alginate/gelatin scaffold. J. Biomed. Mater. Res. A.

[133] Brun F., Turco G., Accardo A., Paoletti S. (2011). Automated quantitative characterization of alginate/hydroxyapatite bone tissue engineering scaffolds by means of micro-CT image analysis. J. Mater. Sci. Mater. Med..

[134] Kolambkar Y.M., Dupont K.M., Boerckel J.D., Huebsch N., Mooney D.J., Hutmacher D.W., Guldberg R.E. (2011). An alginate-based hybrid system for growth factor delivery in the functional repair of large bone defects. Biomaterials.

[135] Nguyen T.P., Lee B.T. (2012). Fabrication of oxidized alginate-gelatin-BCP hydrogels and evaluation of the microstructure, material properties and biocompatibility for bone tissue regeneration. J. Biomater. Appl..

[136] Tan H., Shen Q., Jia X., Yuan Z., Xiong D. (2012). Injectable nano-hybrid scaffold for biopharmaceuticals delivery and soft tissue engineering. Macromol. Rapid Commun..

[137] Cao Y., Shen X.C., Chen Y., Guo J., Chen Q., Jiang X.Q. (2005). pH-Induced self-assembly and capsules of sodium alginate. Biomacromolecules.

[138] Mi F.L., Shyu S.S., Linc Y.M., Wuc Y.B., Peng C.K., Tsai Y.H. (2003). Chitin/PLGA blend microspheres as a biodegradable drug delivery system: A new delivery system for protein. Biomaterials.

[139] Abbah S.A., Liu J., Lam R.W., Goh J.C., Wong H.K. (2012). *In vitro* bioactivity of rhBMP-2 delivered with novel polyelectrolyte complexation shells assembled on an alginate microbead core template. J. Control. Rel..

[140] Zhao Q., Mao Z., Gao C., Shen J. (2006). Assembly of multilayer microcapsules on CaCO_3_ particles from biocompatible polysaccharides. J. Biomater. Sci. Polym. Ed..

[141] Wong Y.Y., Yuan S., Choong C. (2011). Degradation of PEG and non-PEG alginate-chitosan microcapsules in different pH environments. Polym. Degrad. Stabil..

[142] Huang S.H., Hsueh H.J., Jiang Y.L. (2011). Light-addressable electrodeposition of cell-encapsulated alginate hydrogels for a cellular microarray using adigital micromirror device. Biomicrofluidics.

[143] Li H., Leulmi R.F., Juncker D. (2011). Hydrogel droplet microarrays with trapped antibody-functionalized beads for multiplexed protein analysis. Lab Chip..

[144] Meli L., Jordan E.T., Clark D.S., Linhardt R.J., Dordick J.S. (2012). Influence of a three-dimensional, microarray environment on human Cell culture in drug screening systems. Biomaterials.

[145] Sugaya S., Kakegawa S., Fukushima S., Yamada M., Seki M. (2012). Micropatterning of hydrogels on locally hydrophilized regions on PDMS by stepwise solution dipping and *in situ* gelation. Langmuir.

[146] Liu X., Ma L., Mao Z., Gao C. (2011). Chitosan-based biomaterials for tissue repair and regeneration. Adv. Polym. Sci..

